# Fractional order differential equations for chronic liver cirrhosis with frequent hospitalization

**DOI:** 10.1186/s13104-022-06223-9

**Published:** 2022-10-22

**Authors:** Lemesa Bedjisa Dano, Koya Purnachandra Rao, Temesgen Duressa Keno

**Affiliations:** Department of Mathematics, Wollega University, Nekemte, Ethiopia

**Keywords:** Decompensated cirrhosis, Hepatitis B infection, Caputo fractional order, Stability analysis, Heavy alcohol consumption

## Abstract

**Objective:**

Liver cirrhosis, which is considered as the terminal stage of liver diseases, has become life-threatening among non-communicable diseases in the world. Viral hepatitis (hepatitis B and C) is the major risk factor for the development and progression of chronic liver cirrhosis. The asymptomatic stage of cirrhosis is considered as the compensated cirrhosis whereas the symptomatic stage is considered as decompensated cirrhosis. The latter stage is characterized by complex disorder affecting multiple systems of liver organ with frequent hospitalization. In this paper, we formulate system of fractional differential equations of chronic liver cirrhosis with frequent hospitalization to investigate the dynamics of the disease. The fundamental properties including the existence of positive solutions, positively invariant set, and biological feasibility are discussed. We used generalized mean value theorem to establish the existence of positive solutions. The Adams-type predictor-evaluate-corrector-evaluate approach is used to present the numerical scheme the fractional erder model.

**Results:**

Using the numerical scheme, we simulate the solutions of the fractional order model. The numerical simulations are carried out using MATLAB software to illustrate the analytic findings. The analysis reveals that the number of decompensated cirrhosis individuals decreases when the progression rate and the disease’s past states are considered.

**Supplementary Information:**

The online version contains supplementary material available at 10.1186/s13104-022-06223-9.

## Introduction

Liver cirrhosis is a terminal stage of liver scarring (fibrosis) caused by many forms of liver diseases, predominantly viral hepatitis (hepatitis B and C) and chronic alcoholism. Moreover, any forms of liver diseases is considered as a source for liver cirrhosis. Cirrhosis is a significant health problem worldwide as it leads to 1.34 million deaths every year [1]. In clinical terms, compensated cirrhosis and decompensated cirrhosis are the two common stages of cirrhosis. The compensated cirrhosis and decompensated cirrhosis are considered as asymptomatic and symptomatic stages, respectively. Decompensated cirrhosis can regress to a compensated stage in case the etiology of the liver disease is resolved. The disease gradually develops from a compensated cirrhosis to a decompensated cirrhosis and the complications of which often result in hospitalization [2]. In the initial stage, cirrhosis is compensated and patients are asymptomatic at this stage. However, decompensation in patients with compensated cirrhosis is the first occurrence of ascites, oesophageal variceal bleeding, hepatic encephalopathy where frequent hospitalisation is a major point of concern [3, 4].

Many scholars have developed mathematical models and used optimal control strategies to control the spread of infectious diseases. For examples, Din et al. [5] studied the viral dynamics of HBV epidemic model using optimal control theory to control the spread of disease. Khan et al. [6] presented the impact of migration, vaccination and hospitalization rates in the HBV model to investigate the dynamic behavior of the disease. In [7], the authors modeled the transmission dynamics of HBV infection and then extended the model to optimal control problems with vaccination and treatment control strategies. The authors in [8–11] developed mathematical models and applied optimal control theory to eliminate the HBV in the community.

All the above-formulated models were classical differential equations used to present the dynamic behavior of the infectious disease in which history, memory and hereditary properties of the disease are missed. Fractional order differential equations, used to describe realistic situations better than classical models, involve memory effects which occur in most biological models. Morever, fractional order models are powerful tools to address these properties. Recently, fractional differential equations (FDEs) are used to study dynamic behavior of real world problems in ecology (see [12]), biology (see [13–15]), engineering (see [16, 17]) and other fields of applied sciences. Fractional order derivative is used for dealing with any order of derivative and is the promotion of integer derivatives. More precisely, fractional order models are the generalization of the classical order models.

Fractional order models are gaining attentions by many scholars including mathematicians and researchers to study the dynamics of diseases with memory and hereditary effects. For examples, Gul et al. [18] considered a new mathematical model for HBV with asymptomatic carrier class to analyze the dynamics of the disease via Caputo fractional derivative. Simelane et al. [19] developed a three compartmental fractional order HBV model with saturated incidence rate. The authors in [20] studied the global stability of a fractional order model for hepatitis B infection. Shah et al. [21] studied HBV model with treatment via Atangana-Baleanu operator. In [22, 23], the authors studied the dynamics of hepatitis B epidemic model using Caputo fractional and Atangana-Baleanu Caputo derivatives, respectively to present the dynamic behavior of HBV infection. Ullah et al. [24] developed a fractional order derivative for HBV epidemic model with hospitalization class. The authors in [25], suggested that the Caputo fractional operator permits the use of standard initial conditions in terms of derivatives of integer order.

Moreover, the literature reveals that the two infectious stages of HBV have major effects on the transmission and control of the disease. Chronic hepatitis (hepatitis B and C) is one of the world’s leading causes of liver disease and is gradually progressive to cirrhosis. For the first time, the authors in [26] developed a mathematical model of liver fibrosis using a system of partial differential equations to explore the efficacy of potential drugs used to block the progression of liver fibrosis. Dano et al. [27] developed a nonlinear deterministic model to investigate the combined effect of hepatitis B infection and heavy alcohol consumption to discuss the progression dynamics of liver cirrhosis. Hence, we propose a Caputo-type fractional derivative for chronic liver cirrhosis with frequent hospitalization to investigate the rapid progression of the disease. To do this, we will improve and extend Khatun et al. [28] by incorporating the decompensated cirrhosis and hospitalization compartments using Caputo fractional derivative of arbitrary order.

## Main text

## Formulation of the fractional order model

Khatun et al. [28] introduced the application of optimal control theory to reduce the chronic liver cirrhosis incidence by preventing the HBV infection using vaccination and treatment strategies. According to [28], the whole population was divided into five classes such as susceptible, exposed, acute infection, liver cirrhotic and recovered individuals. Based on this, we developed a chronic liver cirrhosis model via Caputo derivative to investigate the memory effects and hereditary factors that influences the dynamics of liver cirrhosis progression. For this purpose, we subdivide the whole population into six compartments. The compartments include susceptible population *S*(*t*), acute infection population *A*(*t*), compensated cirrhosis population *C*(*t*), decompensated cirrhosis population *D*(*t*), hospitalization population *H*(*t*) and recovered population *R*(*t*). In addition, we assumed that the total population is given by $T(t)=S(t)+A(t)+C(t)+D(t)+H(t)+R(t)$. Additional file 1: Fig. S1 represents the flowchart diagram for the transfer between the compartments for chronic liver cirrhosis with frequent hospitalization model. For the sake of simplicity, let

1 $$\begin{aligned} & k_{1}= d_{0}= k_{6}, \quad k_{2}= (d_{0}+\eta _{1}+\eta _{2}+\sigma ),\\ & k_{3} = (d_{0}+d_{1}+s_{1}+s_{2}+s_{3}), \quad k_{4} = (d_{0}+d_{2}+\vartheta _{2}+\mu ),\\ & k_{5} = (d_{0}+d_{3}+\vartheta _{1}+\delta )\end{aligned}$$

Thus, the deterministic model for the dynamics of chronic liver cirrhosis with frequent hospitalization will take the form

2 $$\begin{aligned} {\left\{ \begin{array}{ll} \frac{d}{dt}{S(t)} = b-\beta (A(t)+\gamma _{1} C(t)+\gamma _{2} D(t)) S(t)-k_{1} S(t),\\ \frac{d}{dt}{A(t)} = \beta (A(t)+\gamma _{1} C(t)+\gamma _{2} D(t)) S(t)-k_{2} A(t),\\ \frac{d}{dt}{C(t)} = \eta _{1} A(t)-k_{3} C(t),\\ \frac{d}{dt}{D(t)} = s_{2} C(t)+\vartheta _{1} H(t)-k_{4} D(t),\\ \frac{d}{dt}{H(t)} = \sigma A(t)+s_{3} C(t)+\vartheta _{2} D(t)-k_{5} H(t),\\ \frac{d}{dt}{R(t)} = \eta _{2} A(t)+s_{1} C(t)+\mu D(t)+\delta H(t)-k_{6} R(t) \end{array}\right. } \end{aligned}$$

along the subsidiary conditions

3 $$\begin{aligned}&S(0) = S_{0} \ge 0, \,\, A(0) = A_{0} \ge 0, \,\, C(0) = C_{0} \ge 0, \\ & D(0) = D_{0} \ge 0, \,\, H(0) = H_{0} \ge 0 \,\, \text {and} \,\, R(0) = R_{0} \ge 0 \end{aligned}$$

Basically, the integer order model is the special case of fractional order models where solution of fractional system converges to the solution of integer order system as the order of derivative tends to integer value. Hence, the above initial value problem can be generalized into a fractional order model in Caputo sense as

4 $$\begin{aligned} {} {\left\{ \begin{array}{ll} {^{C} D_{t}^{\alpha }}{S(t)}= b-\beta (A(t)+\gamma _{1} C(t)+\gamma _{2} D(t)) S(t)-k_{1} S(t),\\ {^{C} D_{t}^{\alpha }}{A(t)} = \beta (A(t)+\gamma _{1} C(t)+\gamma _{2} D(t)) S(t)-k_{2} A(t),\\ {^{C} D_{t}^{\alpha }}{C(t)} = \eta _{1} A(t)-k_{3} C(t),\\ {^{C} D_{t}^{\alpha }}{D(t)} = s_{2} C(t)+\vartheta _{1} H(t)-k_{4} D(t),\\ {^{C} D_{t}^{\alpha }}{H(t)} = \sigma A(t)+s_{3} C(t)+\vartheta _{2} D(t)-k_{5} H(t),\\ {^{C} D_{t}^{\alpha }}{R(t)} = \eta _{2} A(t)+s_{1} C(t)+\mu D(t)+\delta H(t)-k_{6} R(t) \end{array}\right. } \end{aligned}$$

with nonnegative initial conditions (IC), where $\alpha \in (0,1], (S,A,C,D,H,R) \in {\mathbb {R}}_{+}^{6}, N=S+A+C+D+H+R$ and if $\alpha =1$, then the fractional order model (4) reduces to the classical order model (2)-(3). Table 1 summarizes the biological description of model parameter and parameter’s value used in the model (4).

**Table 1:** Parameters and parameter values used in the proposed fractional order model

Parameter	Description	Value
*b*	Birth rate	0.0400
$d_{0}$	Natural death rate	0.0100
$d_{1}$	Compensated cirrhotic-related death rate	0.0020
$\sigma$	Hospitalization rate of acute infection	0.0100
$\beta$	Transmission rate	0.0420
$d_{2}$	Decompensated cirrhotic related death rate	0.0020
$\gamma _{1}$	Reduction rate of compensated cirrhosis	0.0020
$\vartheta _{1}$	Decompensation rate of hospitalized population	0.1000
$\vartheta _{2}$	Hospitalization rate of decompensated cirrhosis	0.0100
$\gamma _{2}$	Reduction rate of decompensated cirrhosis	0.0020
$\mu$	Recover rate of decompensated cirrhosis	0.0010
$\delta$	Recover rate of hospitalization individuals	0.0200
$d_{3}$	Hospitalized-related death rate	0.0010
$s_{1}$	Recover rate of compensated cirrhosis	0.1000
$s_{2}$	Decompensation rate	0.2000
$s_{3}$	Hospitalization rate of compensated cirrhosis	0.0200
$\eta _{1}$	Rrogression rate of compensated cirrhosis	0.0200
$\eta _{2}$	Spontaneous recovery rate	0.1500

### Preliminaries

This preliminary part presents the basic definitions commonly used in Caputo-type fractional order models.

#### Definition 1

The fractional integral of order $\alpha > 0$ for a function $x \in L^{1} \left( [0,b], {\mathbb {R}}^{+} \right)$ is defined in the sense of Riemann-Liouville as

5 $$\begin{aligned} {} l^{\alpha }{x(t)} = \frac{1}{\Gamma (\alpha )} {\int _{0}^{t}{(t-s)^{\alpha -1} x(s)}ds}, \end{aligned}$$

where $[0,b] \subseteq {\mathbb {R}}^{+}$ and the Euler’s Gamma function, denoted by $\Gamma (\cdot )$, is defined by

$$\begin{aligned} \Gamma (\alpha ) = \int _{0}^{\infty } {t^{\alpha -1} e^{-t} dt} \end{aligned}$$

for all time $t>0$.

#### Definition 2

The Caputo fractional derivative of order $\alpha > 0$ for function $x :C^{n}[a,b] \rightarrow {\mathbb {R}}$ and $n-1< \alpha < n, ~n \in {\mathbb {Z}}^{+}$ is defined by

6 $$\begin{aligned} {} {^{C}{D_{t}^{\alpha }}{x(t)}} = \frac{1}{\Gamma (n-\alpha )}{\int _{0}^{t}{(t-s)^{n-\alpha -1}x^{(n)}(s)}ds} \end{aligned}$$

which is absolutely continuous function. Basic properties of Caputo derivatives include

(i) $$\displaystyle {^{C}{D_{t}^{\alpha }}}{l^{\alpha }}{x(t)}=x(t)$$
(ii) $$\displaystyle {l^{\alpha }}{^{C}{D_{t}^{\alpha }}}{x(t)}=x(t)-\sum _{j=0}^{n-1}{\frac{x^{(j)}(a)}{j!}(t-a)^{j}}, ~~t>a$$

In particular, the Caputo derivative of order $\alpha \in (0,1)$ for a function $x :C[a,b] \rightarrow {\mathbb {R}}$ is given by

$$\begin{aligned} {^{C}{D_{t}^{\alpha }}{x(t)}} = \frac{1}{\Gamma (1-\alpha )}{\int _{0}^{t}{(t-s)^{-\alpha }x'(s)}ds} \end{aligned}$$

#### Definition 3

Suppose that a constant $x^{*}$ represents the fixed point of system (4). Then the solution of the Caputo fractional model is given by

7 $$\begin{aligned} {} {^{C}{D_{t}^{\alpha }}{x(t)}} = f(t,x(t)), \quad 0 < \alpha \le 1 \end{aligned}$$

if and only if $f(t,x^{*}(t))=0$.

#### Lemma 1

Suppose that $x^{*} \in {\mathbb {D}} \subseteq {\mathbb {R}}^{+}$ is an equilibrium point of system (4). Let $f :[0, \infty ] \rightarrow {\mathbb {R}}$ be continuously differentiable function with

$$\begin{aligned} K_{1} x(t) \le f(t,x(t)) \le K_{2} x(t) \end{aligned}$$

and

$$\begin{aligned} {^{C}{D_{t}^{\alpha }}{f(t,x(t))}} \le -K_{3} x(t) \end{aligned}$$

where $K_{i} x(t)$ represents the positive definite functions for all $i=1,2,3$ which is continuous on $\Omega$. Thus, the fixed point $x^{*}$ is known to be uniformly asymptotically stable equilibrium of system (4) for all $\alpha \in (0,1]$ and $x \in \Omega$.

#### Lemma 2

Suppose that $x(t) \in C[a,b]$ and let $~ _{0}^{C}{D_{t}^{\alpha }}x(t) \in C[a,b]$ and $\alpha \in (0,1]$. We have

$$\begin{aligned} x(t) = x(a)+\frac{1}{\Gamma (\alpha )}{_{0}^{C}{D_{t}^{\alpha }}x(\eta )}{(t-a)^{\alpha }}, \end{aligned}$$

where $$0 < \eta \le 1$$ for $$a \le t \le b$$.

#### Remark 1

Let ${^{C}}{D_{t}^{\alpha }}x(t) \in C[a,b]$ be the Caputo derivative of *x* for $0 < \alpha \le 1$. If ${^{C}}{D_{t}^{\alpha }}x(t) \ge 0$, then $x(t) \in C[a,b]$ is non-decreasing for $a<t<b$ and if ${^{C}}{D_{t}^{\alpha }}x(t) \le 0$, then $x(t) \in C[a,b]$ is non-increasing for $a<t<b$

### Positivity of solutions

To determine the future behavior of the chronic liver cirrhosis, we determine the positivity and existence of solutions of the fractional order model. Consider a closed set

8 $$\begin{aligned} {} \Omega = \{x(t) \in {\mathbb {R}}_{+}^{6} :x(t) \ge 0 \,\, \text {and} \,\, x(t) = \{S(t), A(t), C(t), D(t), H(t), R(t) \}\} \end{aligned}$$

#### Theorem 1

Given a non-negative initial data $x(0)>0$ of system (4). Then the solution *x*(*t*) in $\Omega$ is always non-negative for all $t>0$. Furthermore, assume that

$$\begin{aligned} \lim _{t \rightarrow \infty }{\sup T(t)}=\frac{b}{d_{0}} \end{aligned}$$

#### Proof

Let $\psi (t)=\beta (A(t)+\gamma _{1} C(t)+\gamma _{2} D(t))$. From the first equation of system (4) we have

9 $$\begin{aligned} {} {^{C}{D_{t}^{\alpha }}S(t)} = b-\beta (A(t)+\gamma _{1} C(t)+\gamma _{2} D(t)) S(t)-d_{0} S(t) \end{aligned}$$

Substitution of $\psi (t)$ in Eq. (9), we obtain

10 $$\begin{aligned} {} {^{C}{D_{t}^{\alpha }} S(t)} = b-(\psi (t)+d_{0}) S(t) \end{aligned}$$

Eq. (10) can be rearranged as

11 $$\begin{aligned} {} {^{C}{D_{t}^{\alpha }}} \left[ S(t)e^{\{{d_{0}t+\int _{0}^{t}{\psi (\tau )}d \tau } \} } \right] = b \left[ e^{ \{{d_{0}t+\int _{0}^{t}{\psi (\tau )}d \tau } \} } \right] \end{aligned}$$

Upon simplification of Eq. (11) yields

12 $$\begin{aligned} {} S(t) = e^{-\{d_{0}t+\int _{0}{t}{\psi (\tau )d \tau }\}} \left[ S_{0}+\int _{0}^{t} \left( b e^{ \{{d_{0}x+\int _{0}^{x}{\psi (s)}d s} \} } \right) dt \right] \end{aligned}$$

$$\square$$

Similarly, it can be shown that $x(t)>0$ for all time $t>0$. Recall, the total population is the sum of all non-negative states of system (4) which is given by

13 $$\begin{aligned} {} {^{C}{D_{t}^{\alpha }}T(t)} = b-d_{0} T(t)-(d_{1} C(t)+d_{2} D(t)+d_{3} H(t)) \end{aligned}$$

Without loss of generality Eq. (13) becomes

14 $$\begin{aligned} {} b-d_{0} T(t)-(d_{1} C(t)+d_{2} D(t)+d_{3} H(t)) \le {^{C}{D_{t}^{\alpha }}T(t)} \le b-d_{0} T(t) \end{aligned}$$

Further, it follows that

15 $$\begin{aligned} {} \frac{d_{0}}{(d_{0}+d_{1}+d_{2}+d_{3})} \le \lim _{t \rightarrow \infty }{\inf T(t)} \le \lim _{t \rightarrow \infty }{\sup T(t)} \le \frac{b}{d_{0}} \end{aligned}$$

Hence, the fractional order model for chronic liver cirrhosis is meaningful in the biological feasible region whenever

16 $$\begin{aligned} {} \lim _{t \rightarrow \infty }{\sup T(t)} \le \frac{b}{d_{0}} \end{aligned}$$

Therefore, the closed set

$$\begin{aligned} \Omega = \bigg \{(S,A,C,D,H,R) \in {\mathbb {R}}_{+}^{6} :S,A,C,D,H,R \ge 0 ~\text {and} ~T(t) \le \frac{b}{d_{0}} \bigg \} \end{aligned}$$

is biological invariant region of the fractional order model (4).

### Disease free-equilibrium $({\mathscr {E}}_{0})$

To calculate the expression for the disease free-equilibrium (DFE) point of the fractional order model (4), we set ${^{C} D_{t}^{\alpha }}{S(t)}={^{C} D_{t}^{\alpha }}{A(t)}={^{C} D_{t}^{\alpha }}{C(t)}={^{C} D_{t}^{\alpha }}{D(t)}={^{C} D_{t}^{\alpha }}{H(t)}={^{C} D_{t}^{\alpha }}{R(t)}=0$ and solve the result simultaneously when $S_{0}> 0 ~\text {and}~ A_{0} = C_{0} = D_{0} = H_{0} = R_{0} > 0$. Here, we denote DFE $\displaystyle {\mathscr {E}}_{0} = (S_{0}, A_{0},C_{0}, D_{0}, H_{0}, R_{0})$ where

17 $$\begin{aligned} {} {\mathscr {E}}_{0} = \left( \frac{b}{d_{0}},0,0,0,0,0 \right) \end{aligned}$$

The expression for the basic reproduction number $({\mathscr {R}}_{0})$ is obtained by using the next-generation matrix method [29]. Let $\displaystyle \frac{dx}{dt}=F(x)-V(x)$ where $x=(A,C,D,H)$, $F ~\text {and}~V$ denote the jacobian matrices of the new appearance of transmission rate and the transition rate between compartments evaluated at DFE of system (3) is given by

$$\begin{aligned} F = \left( \begin{array}{cccc} \beta S_{0} &{} \gamma _{1} \beta S_{0} &{} \gamma _{2} \beta S_{0} &{} 0\\ 0 &{} 0 &{} 0 &{} 0\\ 0 &{} 0 &{} 0 &{} 0 \\ 0 &{} 0 &{} 0 &{} 0 \end{array} \right) \quad \text {and} \quad V = \left( \begin{array}{cccc} k_{2} &{} 0 &{} 0 &{} 0\\ - \eta _{1} &{} k_{3} &{} 0 &{} 0\\ 0 &{} -s_{2} &{} k_{4} &{} -\vartheta _{1}\\ -\sigma &{} -s_{3} &{} -\vartheta _{2} &{} k_{5} \end{array} \right) \end{aligned}$$

Thus, $\displaystyle \rho (F V^{-1})$ is the spectral radius which produces the dominant eigenvalue and it represents the basic reproduction number such that

18 $$\begin{aligned} {} {\mathscr {R}}_{0} = \frac{b \beta ((k_{3}+\eta _{1} \gamma _{1}) (k_{4} k_{5}- \vartheta _{1} \vartheta _{2})+\gamma _{2}( k_{5} \eta _{1} s_{2}+\vartheta _{1}(k_{3} \sigma +\eta _{1} s_{3}))}{ k_{1} k_{2} k_{3} (k_{4} k_{5}- \vartheta _{1} \vartheta _{2}) } \end{aligned}$$

This quantity is used to measure the transmission potential of chronic liver cirrhosis and is defined as the expected number of secondary infectious primarily caused by a single infective in an entirely susceptible population.

### Endemic equilibrium $({\mathscr {E}}^{*})$

An endemic equilibrium (EE) point plays an important role in the study of an epidemiological model. It indicates a constant persistence of disease in the population if it exists and it becomes endemic if at least one infected compartments (i.e., A(t), C(t), D(t)) of system (4) becomes non-zero. For the evaluation of the EE, we substitute ${^{C}}{D_{t}^{\alpha }}{S(t)} = {^{C}}{D_{t}^{\alpha }}{A(t)} = {^{C}}{D_{t}^{\alpha }}{C(t)} = {^{C}}{D_{t}^{\alpha }}{D(t)} = {^{C}}{D_{t}^{\alpha }}{H(t)} = {^{C}}{D_{t}^{\alpha }}{R(t)} = 0$ in the right hand side of system (4). Let us denote ${\mathscr {E}}^{*}=(S^{*}, A^{*}, C^{*}, D^{*}, H^{*}, R^{*})$ to be any arbitrary EE. Thus, solving system (4) at steady states yields

19 $$\begin{aligned}&S^{*} = \frac{b}{k_{1} {\mathscr {R}}_{0}}, \\ & A^{*} = \frac{b k_{1} k_{3} (k_{4} k_{5}-\vartheta _{1} \vartheta _{2})) ({\mathscr {R}}_{0}-1)}{\beta ((k_{3}+\eta _{1} \gamma _{1})(k_{4} k_{5}-\vartheta _{1} \vartheta _{2})+\gamma _{2} (k_{5} \eta _{1} s_{2}+\vartheta _{1} (k_{3} \sigma +\eta _{1} s_{3})))}\\ & C^{*} = \frac{b k_{1} \eta _{1} (k_{4} k_{5}-\vartheta _{1} \vartheta _{2})) ({\mathscr {R}}_{0}-1)}{\beta ((k_{3}+\eta _{1} \gamma _{1})(k_{4} k_{5}-\vartheta _{1} \vartheta _{2})+\gamma _{2} (k_{5} \eta _{1} s_{2}+\vartheta _{1} (k_{3} \sigma +\eta _{1} s_{3})))} \\ & D^{*} = \frac{b k_{1} (k_{5} \eta _{1} s_{2}+\vartheta _{1} (k_{3} \sigma +\eta _{1} s_{3}) ({\mathscr {R}}_{0}-1)}{\beta ((k_{3}+\eta _{1} \gamma _{1})(k_{4} k_{5}-\vartheta _{1} \vartheta _{2})+\gamma _{2} (k_{5} \eta _{1} s_{2}+\vartheta _{1} (k_{3} \sigma +\eta _{1} s_{3})))}\\ & H^{*} = \frac{b k_{1} (k_{4}( k_{3} \sigma +\eta _{1} s_{2})+\eta _{1} \vartheta _{2} s_{2}) ({\mathscr {R}}_{0}-1)}{\beta ((k_{3}+\eta _{1} \gamma _{1})(k_{4} k_{5}-\vartheta _{1} \vartheta _{2})+\gamma _{2} (k_{5} \eta _{1} s_{2}+\vartheta _{1} (k_{3} \sigma +\eta _{1} s_{3})))} \end{aligned}$$

From Eq. (19), we notice that the existence of EE of system (4) depends on ${\mathscr {R}}_{0}$. This implies that a unique EE exists if ${\mathscr {R}}_{0}>1$. Therefore, a positive unique EE exists if and only if $k_{4} k_{5} > \vartheta _{1} \vartheta _{2}$ and ${\mathscr {R}}_{0}>1$.

#### Lemma 3

The proposed fractional order model has a positive endemic equilibrium if ${\mathscr {R}}_{0} > 1$.

## Stability analysis

In this section, we present the local asymptotically stability of disease free-equilibrium point of the model (4). Consider the linearization matrix of system (4) evaluated at ${\mathscr {E}}_{0}$

20 $$\begin{aligned} {} J({\mathscr {E}}_{0}) = \left( \begin{array}{cccccc} -d_{0} &{} -\beta S_{0} &{} -\gamma _{1} \beta S_{0} &{} -\gamma _{2} \beta S_{0} &{} 0 &{} 0 \\ 0 &{} \beta S_{0}-k_{2} &{} \gamma _{1} \beta S_{0} &{} \gamma _{2} \beta S_{0} &{} 0 &{} 0 \\ 0 &{} \eta _{1} &{} -k_{3} &{} 0 &{} 0 &{} 0 \\ 0 &{} 0 &{} s_{2} &{} -k_{4} &{} \vartheta _{1} &{} 0 \\ 0 &{} \sigma &{} s_{3} &{} \vartheta _{2} &{} -k_{5} &{} 0\\ 0 &{} \eta _{2} &{} s_{1} &{} \mu &{} \delta &{} -d_{0} \end{array} \right) \end{aligned}$$

### Theorem 2

The DFE of system (4) is locally asymptotically stable (LAS) if and only if ${\mathscr {R}}_{0} < 1$ and unstable otherwise.

### Proof

To discuss the *LAS* of ${\mathscr {E}}_{0}$ of system (4), it is suffice to show all eigenvalues $\lambda _{i}~(i=1,\dots ,6)$ of Eq. (20) satisfies the condition

21 $$\begin{aligned} {} |arg(\lambda _{i})| > \frac{\alpha \pi }{2} \end{aligned}$$

The characteristic equation associated to Eq. (20) becomes

22 $$\begin{aligned} {} (\lambda +d_{0})(\lambda +k_{6})(\lambda ^{4}+a_{1} \lambda ^{3}+a_{2} \lambda ^{2}+a_{3} \lambda +a_{4})=0, \end{aligned}$$

where $a_{i}~(i=1,\dots ,4)$ will take the following value

$$\begin{aligned}&a_{1} = k_{2}+k_{3}+k_{4}+k_{5}-\beta S_{0},\\ & a_{2} = k_{2} k_{3}+ k_{2} k_{4}+ k_{3} k_{5}+ k_{2} k_{5}+ k_{3} k_{4} + (k_{4} k_{5}-\vartheta _{1} \vartheta _{2})-( k_{3}+ k_{4}+ k_{5}+\eta _{1} \gamma _{1}) \beta S_{0},\\ & a_{3} = (k_{4}+ k_{5})( k_{2} k_{3}-( k_{3}+\eta _{1} \gamma _{1})\beta S_{0})+( k_{2}+ k_{3}-\beta S_{0})( k_{4} k_{5}-\vartheta _{1} \vartheta _{2})\\ & ~~~~~~ -\gamma _{2}(\eta _{1} s_{2}+\vartheta _{1} \sigma ) \beta S_{0}\\ & a_{4} = k_{2} k_{3} (k_{4} k_{5}-\vartheta _{1} \vartheta _{2}) (1-{\mathscr {R}}_{0}) \end{aligned}$$

The real parts of the first two eigenvalues $\lambda _{1}=-d_{0} = \lambda _{6}$ are negative. We see that $|arg(\lambda _{1})|=\pi >\frac{\alpha \pi }{2}$ and $|arg(\lambda _{6})|=\pi >\frac{\alpha \pi }{2}$ for $\alpha \in (0,1]$. Clearly, $a_{i}>0$ if and only if $k_{4} k_{5}>\vartheta _{1} \vartheta _{2}$ and ${\mathscr {R}}_{0}>1$. Hence, the condition $a_{1} a_{2}> a_{3} ~\text {and} ~ a_{1} a_{2} a_{3} > a_{3}^{2}+ a_{1}^{2} a_{4}$ hold. Using the Routh-Hurwitz criteria for fractional order models, all the eigenvalues have negative real parts and satisfy equation (22), provided that the resultant of the fourth order polynomial in Eq. (20) is positive. $\square$

### Theorem 3

Given $\alpha \in (0.1]$ and ${\mathscr {R}}_{0} < 1$. Thus, the DFE of system (4) is globally asymptotically stable (*GAS*) in $\Omega$ and unstable if ${\mathscr {R}}_{0} > 1$

### Proof

Consider a suitable Lyapunov function of the form

23 $$\begin{aligned} {} L(t) = b_{1} A(t)+b_{2} C(t)+b_{3} D(t)+b_{4} H(t), \end{aligned}$$

where $b_{i} ~(i=1,\dots , 4)$ are to be chosen later and by applying the Caputo derivative on Eq. (23), we obtain

24 $$\begin{aligned} {} {^{C}{D_{t}^{\alpha }}{L(t)}} = \, b_{1} {^{C}{D_{t}^{\alpha }}{A(t)}}+b_{2} {^{C}{D_{t}^{\alpha }}{C(t)}}+b_{3} {^{C}{D_{t}^{\alpha }}{D(t)}}+b_{4}{^{C}{D_{t}^{\alpha }}{H(t)}} , \end{aligned}$$

Exploiting Eqs. (4) and (24) gives

$$\begin{aligned} {^{C}{D_{t}^{\alpha }}{L(t)}}&= b_{1} ({\beta (A(t)+\gamma _{1} C(t)+\gamma _{2} D(t)) S(t)-k_{2} A(t)})\\ & \,\, +b_{2} (\eta _{1} A(t)-k_{3} C(t))\\ & \,\, +b_{3} ({s_{2} C(t)+\vartheta _{1} H(t)-k_{4} D(t)})\\ & \,\, +b_{4} (\sigma A(t)+s_{3} C(t)+\vartheta _{2} D(t)-k_{5} H(t)) \end{aligned}$$

After rearrangement of the above equation, we obtain

$$\begin{aligned} {^{C}{D_{t}^{\alpha }}{L(t)}}&\le (b_{1} (\beta S_{0} - k_{2}) + b_{2} \eta _{1} +b_{4} \sigma ) A(t) \,\, + (b_{1} \gamma _{1} \beta S_{0} -b_{2} k_{3} + b_{3} s_{2}+b_{4} s_{3} ) C(t)\\ & \,\, + (b_{1} \gamma _{2} \beta S_{0} - b_{3} k_{4}+b_{4} \vartheta _{2}) D(t) \,\, + (b_{3} \vartheta _{1} -b_{4} k_{5}) H(t) \end{aligned}$$

Choose

$$\begin{aligned} b_{i} = \frac{(k_{4} k_{5}-\vartheta _{1} \vartheta _{2})}{\gamma _{2} \beta S_{0}}, ~~ b_{2}=\frac{\gamma _{1} (k_{4} k_{5}-\vartheta _{1} \vartheta _{2})+k_{5} \gamma _{2} s_{2}+\vartheta _{1} \gamma _{2} s_{3}}{k_{3} \gamma _{2}}, ~~ b_{3} = k_{5}, ~~ b_{4} = \vartheta _{1} \end{aligned}$$

Hence, the Lyapunov function *L*(*t*) is contiunous and positive definite for all $A(t), C(t), D(t), H(t)>0$. Furthermore, it follows that

$$\begin{aligned} {^{C}}{D_{0}^{\alpha }}{L(t)} = (k_{1} k_{2} k_{3} (k_{4} k_{5}- \vartheta _{1} \vartheta _{2}) ) \left( {\mathscr {R}}_{0} - 1 \right) A(t) \end{aligned}$$

Hence, ${^{C}}{D_{0}^{\alpha }}{L(t)} \le 0$ if $k_{4} k_{5}>\vartheta _{1} \vartheta _{2}$ and ${\mathscr {R}}_{0} < 1$ and ${^{C}}{D_{0}^{\alpha }}{L(t)} = 0$ if and only if $A(t)=C(t)=D(t)=H(t)=0$. Thus, the largest compact invariant set $\{(S,A,C,D,H,R) \in {\mathbb {R}}^{6} :{^{C}}{D_{0}^{\alpha }}{L(t)} = 0 \}$ contains the singleton, ${\mathscr {E}}_{0}$. Using the LaSalle’s invariant principle [30], we conclude that the DFE of system (4) is *GAS* in $\Omega$ if ${\mathscr {R}}_{0}<1$. $\square$

In the epidemiological models, the sensitivity analysis plays a significant role to provide an in-depth study of state variables that makes predictions and the models more reliable. To do so, we must identify the relative contribution of each model parameter. Some parameters have positive influence on the basic reproduction number while the other parameters are inversely related to the reproductive number (see, Additional file 2: Fig. S2). A slight decrease in the values of $d_{0} ~\text {and} ~d_{2}$ will increase the basic reproduction number as depicted inAdditional file 2: Fig. S2  (*a*). On the other hand, an increase in the values of $b ~\text {and} ~\beta$ will increase the basic reproduction number as seen inAdditional file 2: Fig. S2  (*c*). Generally, $d_{0} ~\text {and} ~d_{2}$ are inversely related to the basic reproduction number while $b ~\text {and} ~\beta$ are directly related which implies that they have relatively significant influence on the control of the disease.

## Numerical simulation

Finding the exact solution of nonlinear fractional differential equations is a difficulty task manually and hence we rely on numerical methods to approximate solutions. We use Adam–Bashforth–Moulton method of the predictor-evaluate-corrector-evaluate (PECE) approach to solve numerically chronic liver cirrhosis model (4) as outlined in [31]. To this end, consider the fractional initial value problem

25 $$\begin{aligned}{{}^{C}}{D_{t}^{\alpha }}{x(t)} &= f(t,x(t)),\\ x^{(k)}(0) & = x_{0}^{k}, \quad k=0,1,\ldots ,[\alpha ]-1, \end{aligned}$$

where $0 \le t \le T ~\text {and} ~f$ represents the Caputo fractional derivative. The solution *x*(*t*) of Eq. (25) is equivalent to the Volterra integral equation

26 $$\begin{aligned} {} x(t) = \sum _{k=0}^{[\alpha ]-1}{\frac{x_{0}^{k}}{k!}{t^{k}}} + \frac{1}{\Gamma (\alpha )}{\int _{0}^{t}{f(\tau ,x(\tau ))(t-\tau )^{\alpha -1}}d \tau } \end{aligned}$$

Utilizing the Adams-type predictor-corrector approach, we approximate the solution of system (4) and integrate Eq. (26) by taking the step-size $h=\frac{T}{N}, ~t=t_{n}=nh$ and $n=0,1,\dots ,N \in {\mathbb {Z}}^{+}$ where $t \in [0,T]$ [32], the iteration formula for Eq. (26) becomes

27 $$\begin{aligned} x_{h}(t_{n+1}) =&\sum _{k=0}^{n-1}{\frac{x_{0}^{k}}{k!}{t_{n+1}^{k}}} + \frac{h^{\alpha }}{\Gamma (\alpha +2)} {\sum _{j=0}^{n}{a_{j,n+1}}{f(t_{j},x_{h}(t_{j}))}}\\ & + \frac{h^{\alpha }}{\Gamma (\alpha +2)} {{f(t_{n+1},x_{h}^{p}(t_{n+1}))}}, \end{aligned}$$

where

28 $$\begin{aligned} {} a_{j,n+1}= {\left\{ \begin{array}{ll} n^{\alpha +1}-(n-\alpha )(n+1)^{\alpha }, \quad \text {if} ~ j=0,\\ (n-j+2)^{\alpha +1}-2 (n-j+1)^{\alpha +1}\\ ~~~~~~~~ + (n-j)^{\alpha +1}, \qquad \qquad \text {if} ~ 0 < j \le n,\\ 1,\qquad \qquad \qquad \text {if} ~ j=n+1 \end{array}\right. } \end{aligned}$$

and the formula for the predictor value $\displaystyle x^{p}(t_{n+1})$ is given by

29 $$\begin{aligned} {} x_{h}^{p}(t_{n+1}) = \sum _{k=0}^{[\alpha ]-1}{\frac{x_{0}^{k}}{k!}{t_{n+1}^{{k}}}} + \frac{1}{\Gamma (\alpha )}{\sum _{j=0}^{n}{b_{j,n+1}}{f(t_{j},x(t_{j}))}}, \end{aligned}$$

where

$$\begin{aligned} b_{j,n+1} = \frac{h^{\alpha }}{\alpha } \left[ (n-j+1)^{\alpha }-(n-j)^{\alpha } \right] \end{aligned}$$

For $p=\min \{2, \alpha +1\}$, we have an error estimate of the scheme as $x_{h}(t_{p})$ is an approximation of $x(t_{n})$ which is given by

$$\begin{aligned} \max _{j=1,2,\dots ,n} \big |x(t_{n})-x_{h}(t_{p}) \big | = O(h^{p}) \end{aligned}$$

The above numerical scheme is applied to approximate the solution of the nonlinear fractional differential equations in (4) with unit of time per year. For convenience, let $\displaystyle x_{h}^{p}(t_{n+1})=(S_{h}^{p}(t_{n+1}), A_{h}^{p}(t_{n+1}), C_{h}^{p}(t_{n+1}), D_{h}^{p}(t_{n+1}), H_{h}^{p}(t_{n+1}),R_{h}^{p}(t_{n+1}))$ and $x_{h}(t_{j})=(S_{h}(t_{j}), A_{h}(t_{j}), C_{h}(t_{j}), D_{h}(t_{j}), H_{h}(t_{j}),R_{h}(t_{j}))$. Thus,

30 $$\begin{aligned}&S_{h}(t_{n+1}) = S_{0} + \frac{h^{\alpha }}{\Gamma (\alpha +2) }{f_{1} (t_{n+1},x_{h}^{p}(t_{n+1}))} + \frac{h^{\alpha }}{\Gamma (\alpha +2)} \sum _{j=0}^{n}{a_{j,n+1}} {f_{1} (t_{j},x_{h}(t_{j}))}, \\ & A_{h}(t_{n+1}) = A_{0} + \frac{h^{\alpha }}{\Gamma (\alpha +2) } {f_{2} (t_{n+1},x_{h}^{p}(t_{n+1}))} + \frac{h^{\alpha }}{\Gamma (\alpha +2)} \sum _{j=0}^{n}{a_{j,n+1}} {f_{2} (t_{j},x_{h}(t_{j}))},\\ & C_{h}(t_{n+1}) = C_{0} + \frac{h^{\alpha }}{\Gamma (\alpha +2) } {f_{3} (t_{n+1},x_{h}^{p}(t_{n+1}))} + \frac{h^{\alpha }}{\Gamma (\alpha +2)} \sum _{j=0}^{n}{a_{j,n+1}} {f_{3} (t_{j},x_{h}(t_{j}))},\\ & D_{h}(t_{n+1}) = D_{0} + \frac{h^{\alpha }}{\Gamma (\alpha +2) } {f_{4} (t_{n+1},x_{h}^{p}(t_{n+1}))} + \frac{h^{\alpha }}{\Gamma (\alpha +2)} \sum _{j=0}^{n}{a_{j,n+1}} {f_{4} (t_{j},x_{h}(t_{j}))},\\ & H_{h}(t_{n+1}) = H_{0} + \frac{h^{\alpha }}{\Gamma (\alpha +2) } {f_{5} (t_{n+1},x_{h}^{p}(t_{n+1}))} + \frac{h^{\alpha }}{\Gamma (\alpha +2)} \sum _{j=0}^{n}{a_{j,n+1}} {f_{5} (t_{j},x_{h}(t_{j}))},\\ & R_{h}(t_{n+1}) = R_{0} + \frac{h^{\alpha }}{\Gamma (\alpha +2) } {f_{6} (t_{n+1},x_{h}^{p}(t_{n+1}))} + \frac{h^{\alpha }}{\Gamma (\alpha +2)} \sum _{j=0}^{n}{a_{j,n+1}} {f_{6} (t_{j},x_{h}(t_{j}))}. \end{aligned}$$

and the predictor values are given by

31 $$\begin{aligned}&S_{h}^{p}(t_{n+1}) = S_{0} + \frac{1}{\Gamma (\alpha )} \sum _{j=0}^{n}{b_{j,n+1}} {f_{1} (t_{j},x_{h}(t_{j}))}, \\ & A_{h}^{p}(t_{n+1}) = A_{0} + \frac{1}{\Gamma (\alpha )} \sum _{j=0}^{n}{b_{j,n+1}} {f_{2} (t_{j},x_{h}(t_{j}))},\\ & C_{h}^{p}(t_{n+1}) = C_{0} + \frac{1}{\Gamma (\alpha )} \sum _{j=0}^{n}{b_{j,n+1}} {f_{3} (t_{j},x_{h}(t_{j}))},\\ & D_{h}^{p}(t_{n+1}) = D_{0} + \frac{1}{\Gamma (\alpha )} \sum _{j=0}^{n}{b_{j,n+1}} {f_{4} (t_{j},x_{h}(t_{j}))},\\ & H_{h}^{p}(t_{n+1}) = H_{0} + \frac{1}{\Gamma (\alpha )} \sum _{j=0}^{n}{b_{j,n+1}} {f_{5} (t_{j},x_{h}(t_{j}))},\\ & R_{h}^{p}(t_{n+1}) = R_{0} + \frac{1}{\Gamma (\alpha )} \sum _{j=0}^{n}{b_{j,n+1}} {f_{6} (t_{j},x_{h}(t_{j}))} \end{aligned}$$

where

$$\begin{aligned}&f_{1}(t_{n+1},x_{n+1}^{p}) = b-\beta (A^{p}(t_{n+1})+\gamma _{1}C^{p}(t_{n+1})+\gamma _{2}D^{p}(t_{n+1}))S^{p}(t_{n+1})-k_{1}S^{p}(t_{n+1}),\\ & f_{2}(t_{n+1},x_{n+1}^{p}) = \beta (A^{p}(t_{n+1})+\gamma _{1}C^{p}(t_{n+1})+\gamma _{2}D^{p}(t_{n+1}))S^{p}(t_{n+1})-k_{2} A^{p}(t_{n+1}), \\ & f_{3}(t_{n+1},x_{n+1}^{p}) = \eta _{1} A^{p}(t_{n+1})-k_{3} C^{p}(t_{n+1}),\\ & f_{4}(t_{n+1},x_{n+1}^{p}) = s_{2} A^{p}(t_{n+1})+\vartheta _{1} H^{p}(t_{n+1})-k_{4} D^{p}(t_{n+1}), \\ & f_{5}(t_{n+1},x_{n+1}^{p}) = s_{1} C^{p}(t_{n+1})+\vartheta _{2} D^{p}(t_{n+1})-k_{5} H^{p}(t_{n+1}),\\ & f_{6}(t_{n+1},x_{n+1}^{p}) = \eta _{1} A^{p}(t_{n+1})+s_{1} C^{p}(t_{n+1})+\mu D^{p}(t_{n+1})+\delta H^{p}(t_{n+1})-k_{6} R^{p}(t_{n+1}) \end{aligned}$$

Similarly, the functions $f_{i}(t_{j},x(t_{j}))$ for $i=1,\dots ,6$ will take the form

$$\begin{aligned}&f_{1}(t_{j},x(t_{j})) = b-\beta (A(t_{j})+\gamma _{1} C(t_{j})+\gamma _{2} D(t_{j}))S(t_{j})-k_{1} S(t_{j}),\\ & f_{2}(t_{j},x(t_{j})) = \beta (A(t_{j})+\gamma _{1}C(t_{j})+\gamma _{2}(t_{j})) S(t_{j})-k_{2} A (t_{j}), \\ & f_{3}(t_{j},x(t_{j})) = \eta _{1} A(t_{j})-k_{3} C(t_{j}),\\ & f_{4}(t_{j},x(t_{j})) = s_{2} A(t_{j})+\vartheta _{1} H(t_{j})-k_{4} D(t_{j}), \\ & f_{5}(t_{j},x(t_{j})) = \sigma A(t_{j})+s_{3} C(t_{j})+\vartheta _{2} D(t_{j})-k_{5} H(t_{j}),\\ & f_{6}(t_{j},x(t_{j})) = \eta _{2} A(t_{j})+s_{1} C(t_{j})+\mu D(t_{j})+\delta H(t_{j})-k_{6} R(t_{j}) \end{aligned}$$

Now, for the numerical simulation of the chronic liver cirrhosis we use the parameter values listed in Table 1. The value of some parameters are assumed in the biological feasible region and the value of the rest parameters are taken from published papers. For instances, the value of $d_{0},d_{1},\delta , \sigma , \eta _{1} ~\text {and}~\eta _{2}$ are taken from [5, 28]. In addition, we assumed the set of initial value for state variables of system (4) are (50, 30, 20, 10, 5, 0) for *S*(*t*), *A*(*t*), *C*(*t*), *D*(*t*), *H*(*t*), *R*(*t*) respectively.

## Results and discussion

For numerical approximation of chronic liver cirrhosis model solution, we utilize the generalized Adams–Bashforth–Moulton technique and implement with MATLAB r2018a software. Using the parameter values and initial value for state variables with varying order of derivative agrees with the theoretical discussions made, We illustrate the numerical results graphically which verifies the asymptotic stability of DFE and EE points when ${\mathscr {R}}_{0} =0.0291<1$ and ${\mathscr {R}}_{0} = 15.2981 > 1$.

Additional file 3: Fig. S3 represents the numerical solution trajectories of the nonlinear fractional differential equations falling toward zero. Additional file 3: Fig. S3  (*d*) shows the number of decompensated cirrhotic individuals increases when the order of derivative $\alpha$ increases, which implies that the past influence of the disease including viral infections (hepatitis B and C), chronic alcoholism and other unknown etiologies would not be considered sufficiently. As clearly seen in Additional file 3: Fig. S3e, the number of hospitalized individuals decreases if we decrease the past influence of the disease. The collective idea of Additional file 3: Fig. S3 is that it is possible to numerically solve nonlinear fractional order for chronic liver cirrhosis model and all solution trajectories converging to disease free-equilibrium point, ${\mathscr {E}}_{0} = \big (4,0,0,0,0,0 \big )$. Biologically, this mean that the disease wipe out of the population but take long period of time even when ${\mathscr {R}}_{0} = 0.0291<1$ without using any control measure.

The endemic equilibrium point of the proposed fractional order model is obtained to be ${\mathscr {E}}^{*} = (1.3073, 72.2519, 30.7455, 29.4655, 37.4599, 216.9896)$ which indicates that there is a constant positive solution in the passage of time. Additional file 4: Fig. S4 indicates that the solution trajectories are moving toward a positive endemic equilibrium when ${\mathscr {R}}_{0} = 15.2981 > 1$ with different values of order of derivative $\alpha$. In Additional file 4: Fig. S4  (*d*), the number of decompensated cirrhosis individuals increases to the maximum positive EE point. Similarly, when past influences of chronic liver cirrhosis are almost ignored (i.e., $\alpha =0.99$) or short memory effects of the disease is considered, the number of hospitalized individuals increases even if we decrease the hospitalization rate of decompensated cirrhosis as observed from Additional file 5: Fig. S5  (*a*). On the contrary, from Additional file 5: Fig. S5  (*b*) we observe that when past influence of chronic liver cirrhosis is considered (i.e., $\alpha =0.10$) or long memory effects of the disease is taken into account, an increase in $\vartheta _{2}$ will decrease the number of hospitalized decompensated cirrhosis individuals.

## Conclusion

In this paper, a system of fractional differential equations is formulated in the context of liver cirrhosis development and progression. A six compartmental fractional order model of chronic liver cirrhosis is proposed and analyzed. Mathematical analysis of the model including existence of positive solutions, positive invariant set, biological feasibility and stability analysis of the fractional order model are presented. Local stability analysis of multiple equilibrium points of the model are checked using fractional Routh–Hurwitz criteria. Accordingly, both DFE and EE are locally asymptotically stable in the biological feasible region if ${\mathscr {R}}_{0}<1 ~\text {and} ~{\mathscr {R}}_{0}>1$, respectively. We employed the generalized Adams–Bashforth–Moulton method to present the numerical scheme. Using the numerical scheme, we performed a large scale of numerical simulations varying the value of the parameter $\alpha$ (order of derivative) to observe the effects that the parameter has on the dynamics of the proposed fractional-order model. The results of the analysis shows that the two stages of cirrhosis (compensated and decompensated cirrhosis) are significant classes of the model should be given emphasize. Furthermore, cirrhosis is a rapidly progressive among the non-communicable disease. The number of decompensated cirrhosis individuals decreases and the number of hospitalized individuals increases when the value of the hospitalization rate increases for various value order of derivatives.

Indeed, the analysis of this paper is far from complete, therefore, any interested researcher can make an extension of the optimal control theory to block the progression of chronic liver cirrhosis.

### Limitation of the paper

This model was designed to present the development and progression of chronic liver cirrhosis. We considered hepatitis B infection as a major contributor of liver diseases. However, any kinds of liver diseases can be considered as a source for cirrhosis.

## Supplementary Information

**Additional file 1: Figure S1.** Flowchart diagram for chronic liver cirrhosis with frequent hospitalization.

**Additional file 2: Figure S2.** The plots demonstrate the basic reproduction number against (a) $\vartheta _{1} ~\text {and} ~\vartheta _{2}$ (b) $\vartheta _{2} ~\text {and} ~\mu$ and (c) b and $\beta$

**Additional file 3: Figure S3.**Simulation results for (a) S(t),(b) A(t), (c) C(t), (d) D(t), (e) H(t), (f) R(t) with varying value of $\alpha$ when ${\mathscr {R}}_{0} = 0.0291<1$

**Additional file 4: Figure S4.** Simulation results for (a) S(t),(b) A(t), (c) C(t), (d) D(t), (e) H(t), (f) R(t) with varying value of $\alpha$ when ${\mathscr {R}}_{0} = 152.9818 > 1$

**Additional file 5: Figure S5.** Simulation results for hospitalization of decompensated cirrhotic individuals when (a) $\alpha =0.99$ and (b) $\alpha =0.5$ with varying hospitalization rate of decompensed cirrhosis

## Data Availability

The authors declare that the data supporting the findings are included in the paper.

## Abbreviations

DFE: Disease free-equilibrium
EE: Endemic equilibrium
FDEs: Fractional differential equations
LAS: Locally asymptotically stable
GAS: Globally asymptotically stable
CF: Caputo-Fabrizio
